# Dynamic chest radiography for pulmonary vascular diseases: clinical applications and correlation with other imaging modalities

**DOI:** 10.1007/s11604-023-01483-2

**Published:** 2023-08-26

**Authors:** Yuzo Yamasaki, Takeshi Kamitani, Koji Sagiyama, Takuya Hino, Megumi Kisanuki, Kosuke Tabata, Takuro Isoda, Yoshiyuki Kitamura, Kohtaro Abe, Kazuya Hosokawa, Daisuke Toyomura, Shohei Moriyama, Masateru Kawakubo, Hidetake Yabuuchi, Kousei Ishigami

**Affiliations:** 1 Department of Clinical Radiology, Graduate School of Medical Sciences, Kyushu University, 3-1-1 Maidashi, Higashi-Ku, Fukuoka, 812-8582 Japan; 2 Department of Hematology, Oncology and Cardiovascular Medicine, Graduate School of Medical Sciences, Kyushu University, Fukuoka, Japan; 3 Department of Cardiovascular Medicine, Graduate School of Medical Sciences, Kyushu University, Fukuoka, Japan; 4 Department of Pediatrics, Graduate School of Medical Sciences, Kyushu University, Fukuoka, Japan; 5 Department of Health Sciences, Graduate School of Medical Sciences, Kyushu University, Fukuoka, Japan

**Keywords:** Dynamic chest radiography, Novel functional X-ray imaging, Pulmonary perfusion, Readily available, Low radiation exposure, Diagnostic imaging

## Abstract

Dynamic chest radiography (DCR) is a novel functional radiographic imaging technique that can be used to visualize pulmonary perfusion without using contrast media. Although it has many advantages and clinical utility, most radiologists are unfamiliar with this technique because of its novelty. This review aims to (1) explain the basic principles of lung perfusion assessment using DCR, (2) discuss the advantages of DCR over other imaging modalities, and (3) review multiple specific clinical applications of DCR for pulmonary vascular diseases and compare them with other imaging modalities.

**Supplementary Information:**

The online version contains supplementary material available at 10.1007/s11604-023-01483-2.

## Introduction

The assessment of pulmonary perfusion is essential for many pulmonary diseases. Lung perfusion scintigraphy and contrast-enhanced computed tomography (CT) play important roles in these evaluations in clinical practice. However, the use of scintigraphy may be restricted owing to the prerequisite such as large sized facilities and equipment, or the difficulties in managing urgent examinations. Contrast-enhanced CT is a widely available and reliable method for most pulmonary vascular diseases; however, its use is occasionally limited because of the requirement of contrast media or high radiation doses. Dynamic chest radiography (DCR) is a novel imaging technique based on conventional X-ray technology that visualizes pulmonary perfusion without contrast media or radionuclides and requires a small space for installation and short examination time. At times, these benefits of DCR exert compelling effects. The utility of this promising technique has been demonstrated in phantoms, animals, and humans with various pulmonary diseases [1–7]. Thus, evidence regarding DCR perfusion imaging has accumulated. Herein, we review the existing knowledge on DCR and discuss its clinical applications.

### Basic principles of DCR

DCR is a functional chest imaging technique that utilizes sequential images obtained using a flat-panel detector (FPD). Recent technical advances, such as FPD with a large field of view, improved sensitivity of X-ray detectors, and technical advances in computer analysis and image post-processing, have enabled the performance of DCR [8]. Only three equipment, namely a pulsed X-ray generator, an FPD supporting cineradiography, and a dedicated analysis software are required to capture and analyze the DCR images. Therefore, DCR can be performed in a general X-ray examination room and has the potential for use as a portable examination system. During DCR, sequential X-ray images are captured using cineradiography. Synchronized pulsed X-rays are irradiated to the patients at 15 frames per second for 7–15 s. Sequential images are analyzed using a dedicated analysis software (KINOSIS, KONICA MINOLTA, Inc., Japan).

The unique feature of DCR is its positional flexibility, allowing it to be performed in both the standing and supine positions. DCR can be used to evaluate the physiological state as it can be performed in a standing position. Other than DCR, the recently developed upright CT and perfusion scintigraphy with radioisotope injection in the sitting position can provide this information [9, 10]. In contrast, DCR in the supine position has the potential to be an alternative to other imaging modalities, such as CT or scintigraphy, which are performed in the same position.

**Fig. 1:**
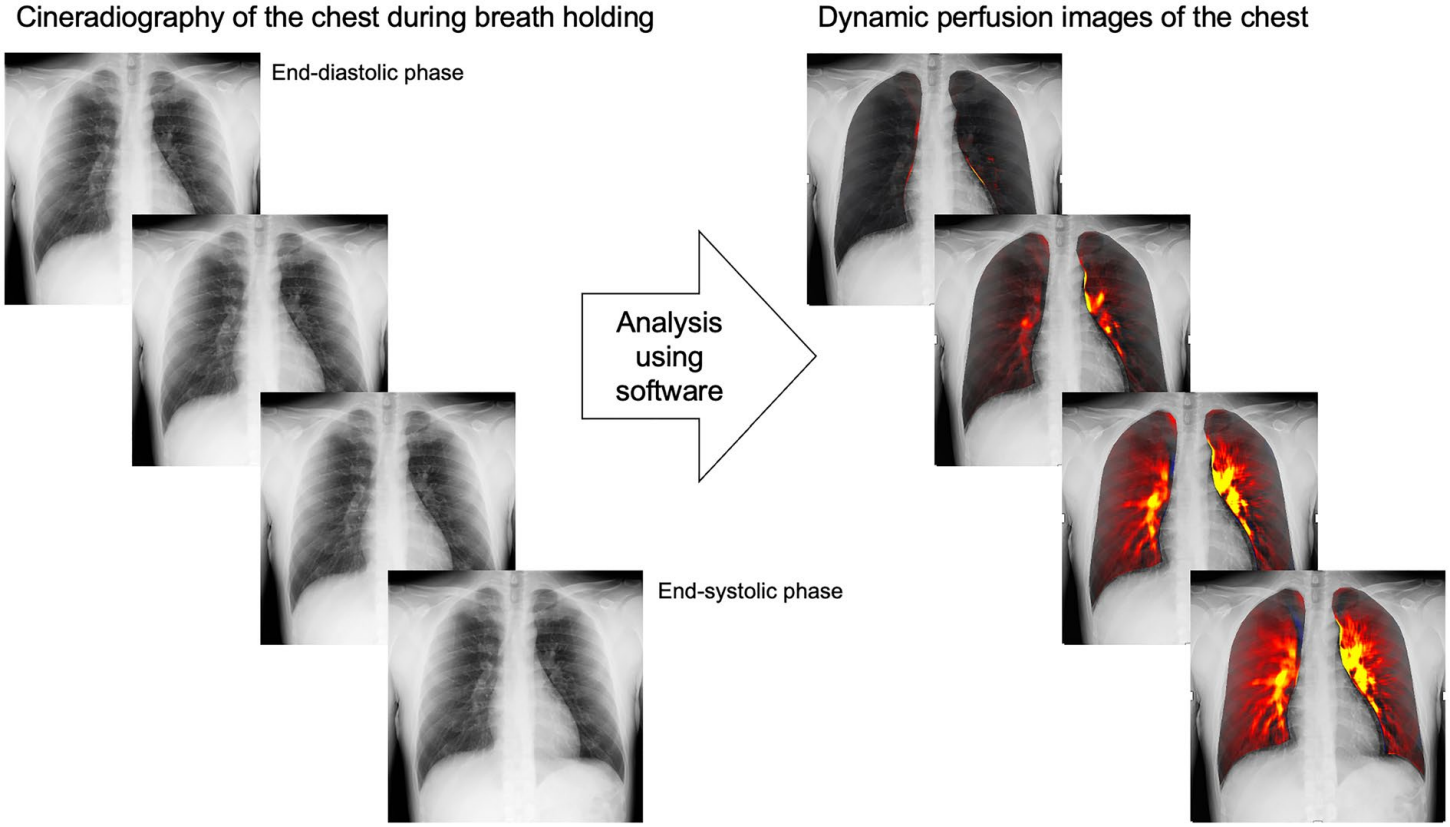
Dynamic perfusion images of the chest created from cineradiography of the chest while holding breath. The increased pulmonary perfusion in each cardiac phase from the end-diastolic phase is visualized in red or yellow by analyzing sequential images

**Fig. 2:**
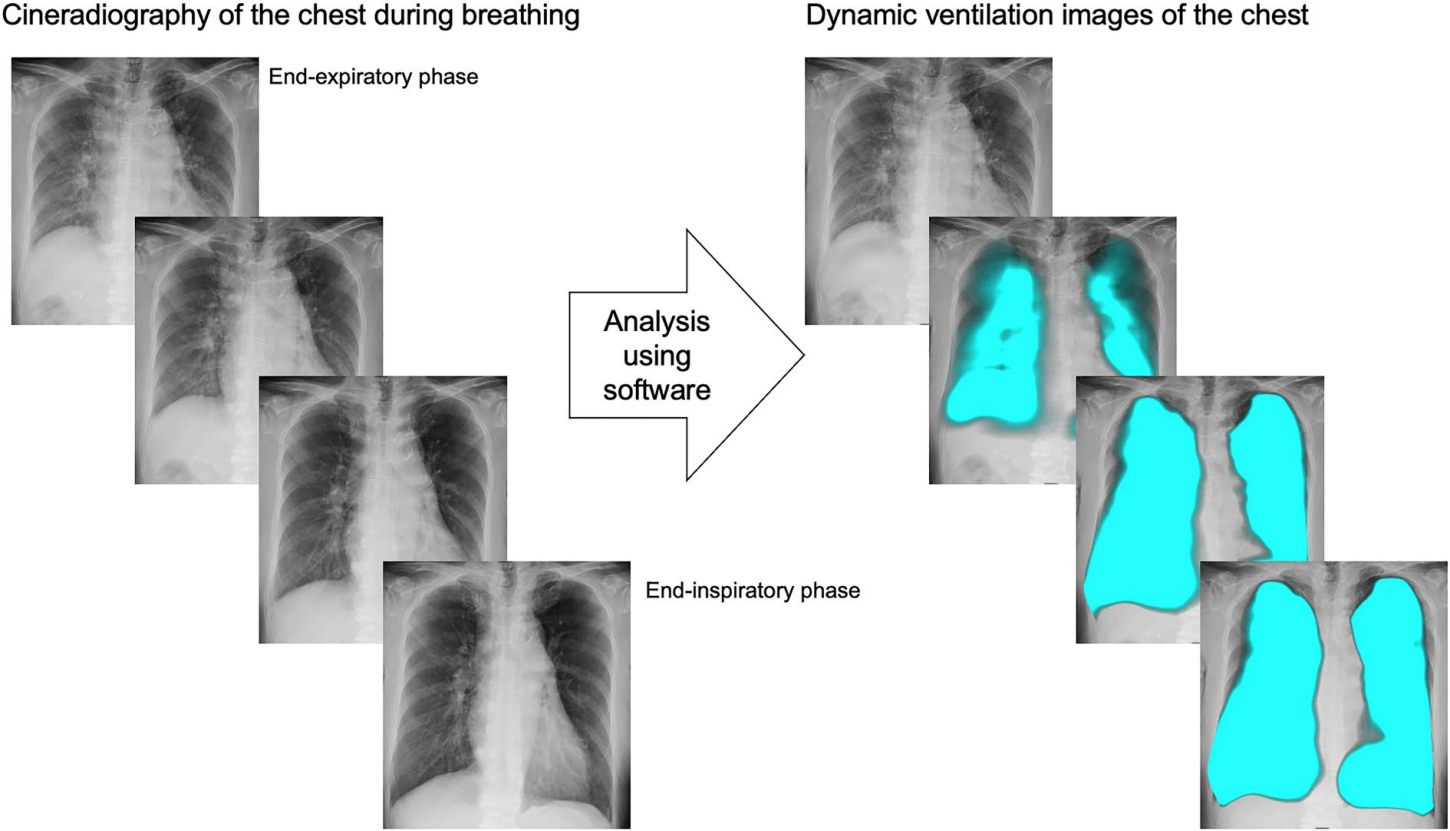
Dynamic ventilation images of the chest created from cineradiography of the chest while breathing. The air filling in the lung during each respiratory phase from the end-expiratory phase is visualized in blue by analyzing the sequential images

DCR can be performed with any breathing style. Perfusion images are created using cineradiography while holding breath (Fig. 1), and ventilation images are created using cineradiography during deep breathing (Fig. 2). The scan time for perfusion imaging is 7–10 s. DCR perfusion images can serve as alternatives to perfusion scintigraphy, contrast-enhanced CT pulmonary angiography (CTPA), and MR angiography in some clinical scenarios. The target diseases include pulmonary embolism (PE), pulmonary hypertension (PH), adult congenital heart disease (ACHD), and pulmonary vascular malformations. In contrast, the scan time for ventilation imaging is 15 s. DCR ventilation imaging can potentially serve as an alternative to pulmonary function testing and ventilation scintigraphy. The target diseases include chronic obstructive pulmonary disease [11], interstitial lung disease [12], lung cancer [13], and diaphragm dysfunction [14]. In this review, we focus on perfusion imaging using DCR and its clinical utility and application.

**Fig. 3:**
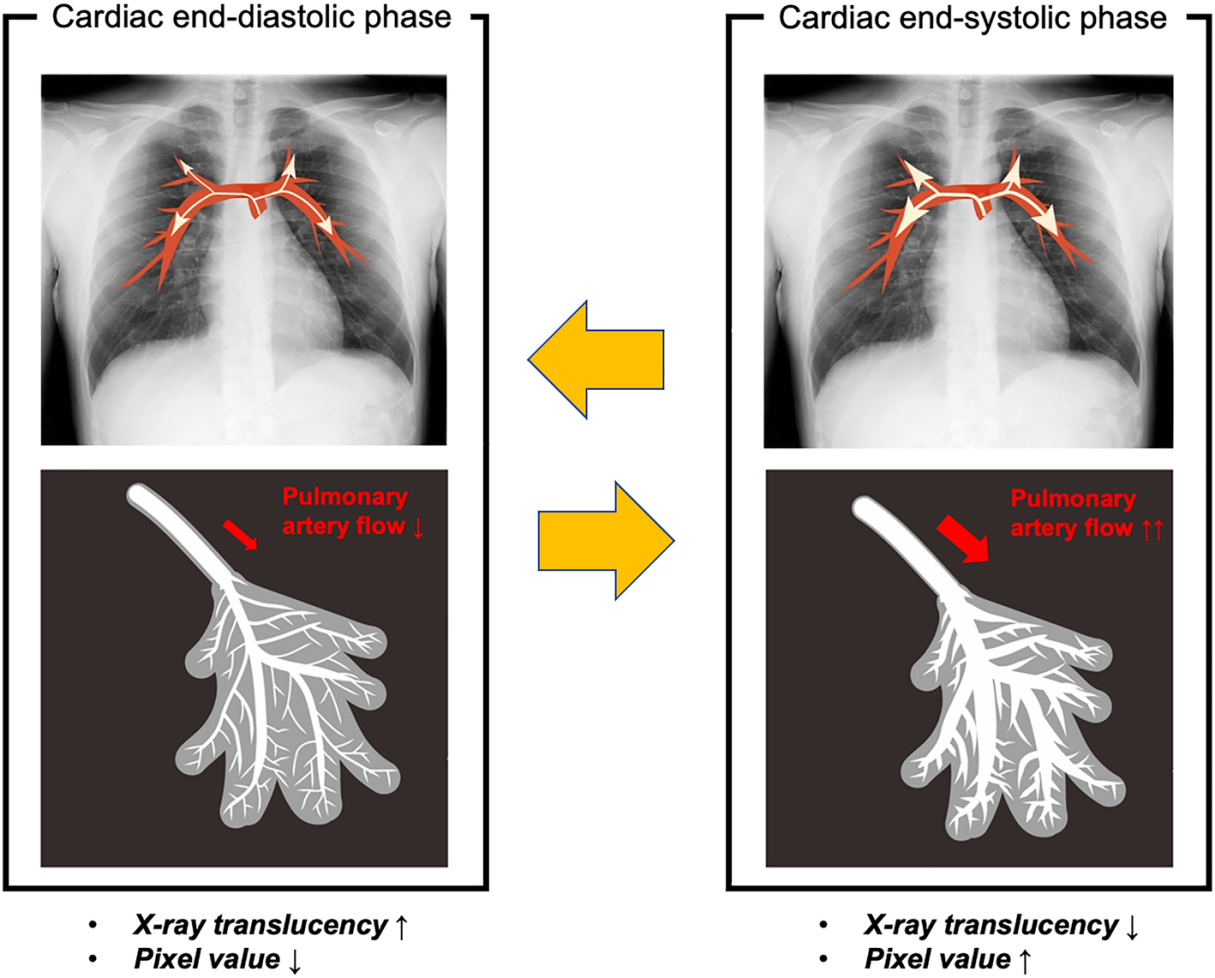
Theory of perfusion imaging of dynamic chest radiography. The pulmonary artery flow increases during the systolic phase, leading to increased blood and vessel volumes in the lungs. This results in a slight temporal decrease in the X-ray translucency. Dynamic chest radiography analyzes small temporal changes in X-ray translucency (pixel value) by cardiac pumping and visualizes pulmonary perfusion. *This figure was created using materials used in Ref 8 (Hata A, Yamada Y, Tanaka R, et al. Dynamic Chest X-Ray Using a Flat-Panel Detector System: Technique and Applications. Korean J Radiol. 2020;21)

The theory of DCR perfusion imaging is based on the changes in X-ray translucency during the cardiac cycle. Sequential images acquired during breath-holding contain information about pulmonary blood flow by cardiac pumping, which is expressed as small temporal changes in X-ray translucency (pixel value) (Fig. 3) [8]. Pulmonary artery flow increases during the systolic phase, resulting in increased blood and vessel volumes in the lungs. Consequently, the X-ray translucency is reduced in the pulmonary vasculature, and the pixel values in the grayscale image (black = 0) increase. The opposite trend is observed during the diastolic phase. These small temporal changes in sequential images are analyzed and visualized as dynamic perfusion images in color. The typical imaging parameters for DCR perfusion imaging are listed in Table 1.

**Table 1:** Machines and typical scan parameters for DCR scanning (body mass index: 25)

Flat-panel detector	AeroDR fine (KONICAMINOLTA, Inc., Japan)	
Pulsed X-ray generator	RADspeed Pro (SHIMADZU, Corp., Japan)	
Position	Standing	Supine
Tube voltage	100 kV	95 kV
Tube current	80 mA	250 mA
Direction	Posteroanterior	Anteroposterior
Frames per second	15	
Duration of pulsed X-ray	7.1 ms	3.2 ms
Additional filter	0.2 mm Cu	0.3 mm Cu
Source to image distance	2 m	1.5 m
Matrix size	1062 × 1062 pixels	
Whole image area	42.5 × 42.5 cm	

^a^Abbreviations: *DCR* dynamic chest radiography

### Analysis methods of DCR lung perfusion imaging

Pulmonary perfusion may be visualized using two types of analysis methods. Both the methods fundamentally utilize temporal changes in pixel values in sequential images obtained by cineradiography of the chest.

#### Cross-correlation analysis method (conventional technique)

**Fig. 4:**
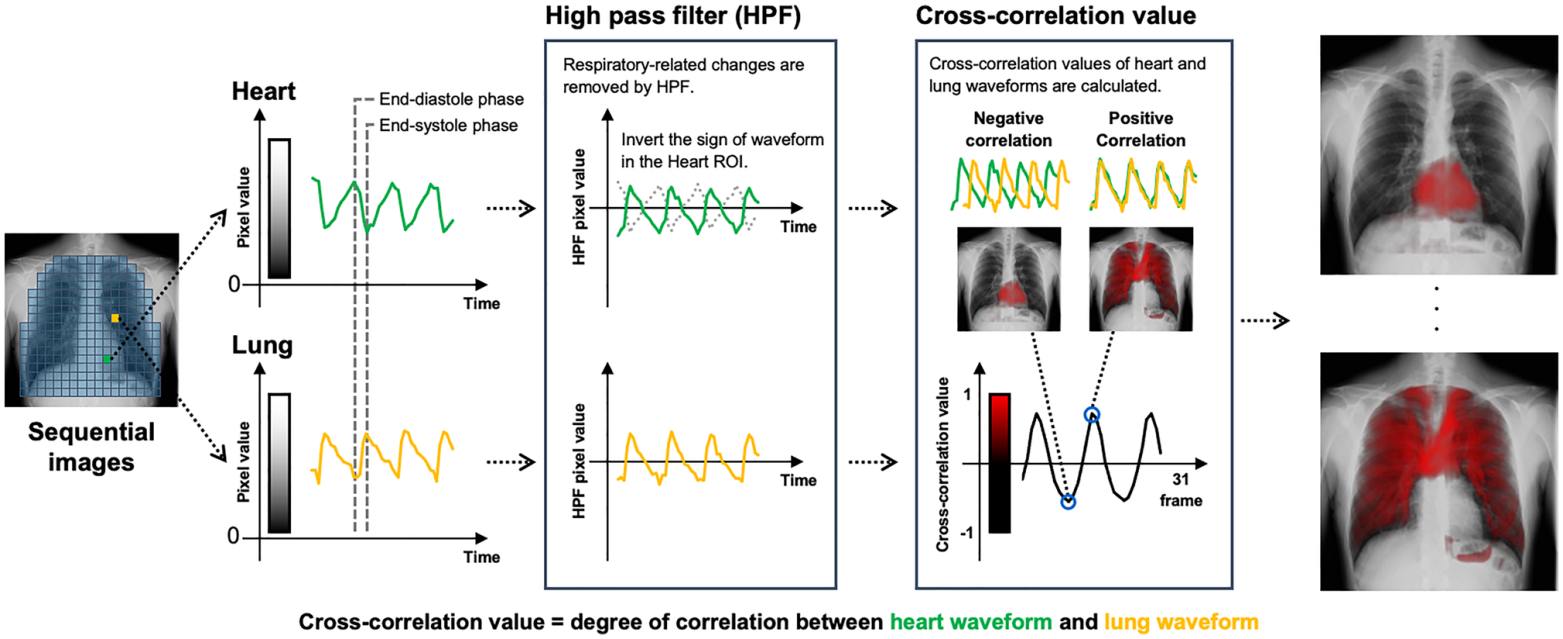
Cross-correlation analysis method of dynamic chest radiography to visualize the pulmonary perfusion. The regions of interest are located in the lungs and heart, and a high-pass filter is applied to both signal waveforms to extract cardiac cycle-related signals. The correlation coefficient between the lung signal curve and inverted heart signal curve is calculated. A large coefficient indicates good consistency between the lung signal, pulmonary artery flow pattern, and preserved perfusion. A perfusion image is obtained as a color map of the spatial distribution of the correlation coefficients. The calculation of the correlation coefficient is repeated by shifting the inverted heart signal curve in the time axis, because it is assumed that the pulmonary artery flow timing varies depending on the location in the lung. Finally, the sequential perfusion images are obtained

The cross-correlation analysis method is based on evaluating the correlation between waveforms in the heart and lungs. After removing the respiratory-related temporal change in pixel values using a high-pass filter, the degree of correlation between the waveforms of pulse-related pixel values in the heart and those in the lungs are analyzed. High correlation is indicated in red. The detailed imaging process and typical images are shown in Fig. 4 and Video 1a. This method offers a robust approach for calculating pulmonary perfusion. Notably, even when the lung waveform exhibits small amplitudes, a high correlation value can be achieved as long as the phase of variation remains correlated. Consequently, this technique enables clear visualization of lung blood vessels, even in regions with subtle blood flow changes.

#### Reference frame subtraction method (advanced technique)

**Fig. 5:**
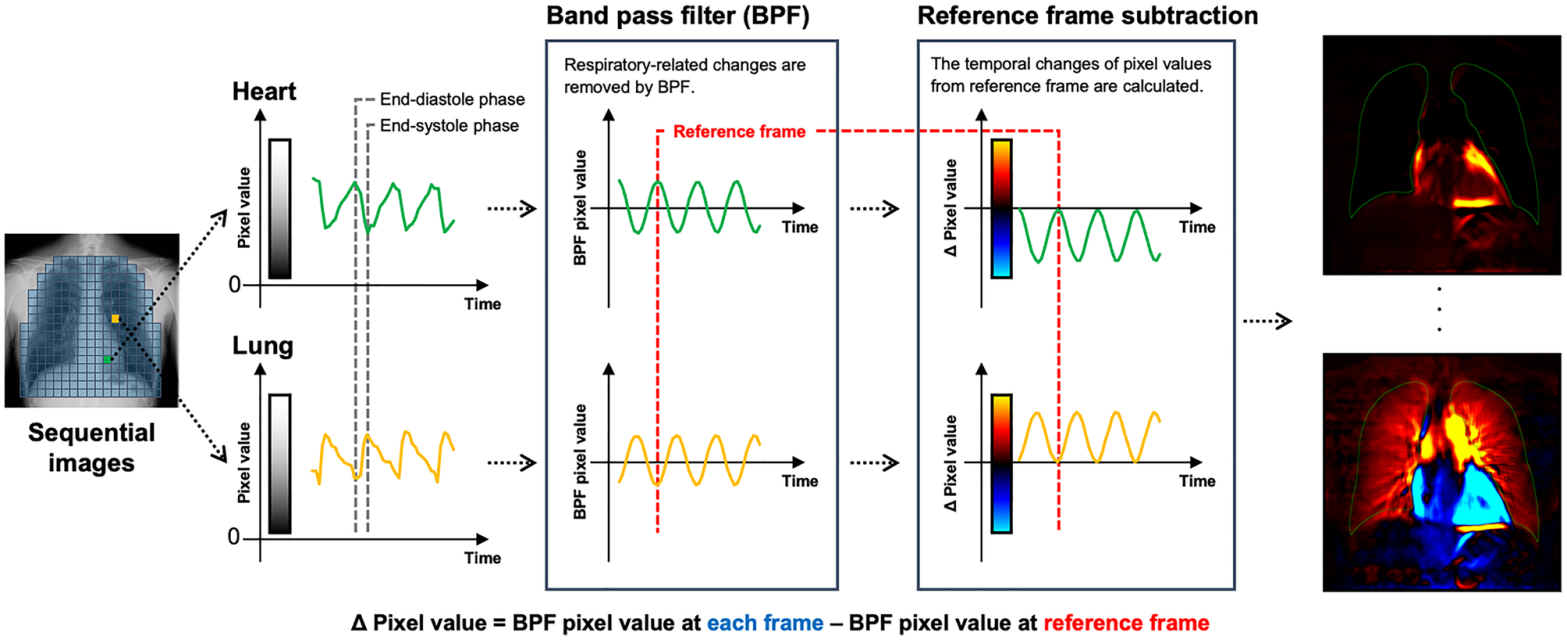
Reference frame subtraction analysis method of dynamic chest radiography to visualize the pulmonary perfusion. The temporal change in the pixel value for each pixel is calculated from sequential images. A band-pass filter is applied to extract cardiac cycle-related signals. The end-diastolic and end-systolic phases are automatically estimated from pixel value changes in the heart. The timing of the highest pixel value, which represents the maximum blood volume in the heart, is defined as the end-diastolic phase. The timing of the lowest pixel value, which represents the minimum blood volume in the heart, is defined as the end-systolic phase. The temporal change in the pixel value from the end-diastolic phase is color-coded and visualized as dynamic perfusion images. Low blood volume is indicated in blue and high blood volume is indicated in red. Black color indicates no change in the interval. Finally, the sequential perfusion images are obtained. *This figure was created using the materials used in Ref 6 (Yamasaki Y, et al. Efficacy of Dynamic Chest Radiography for Chronic Thromboembolic Pulmonary Hypertension. Radiology. 2023; 306(3): e220908)

The reference frame subtraction method visualizes the degree of pixel value change from the baseline timing (end-diastolic phase). After removing the respiratory-related temporal changes in pixel values using a band-pass filter, the interval changes from the reference frame (endo-diastolic phase) at each phase are analyzed. A large interval change in the pixel value is colored red and yellow. The detailed imaging process and typical images are shown in Fig. 5 and Video 1b.

**Fig. 6:**
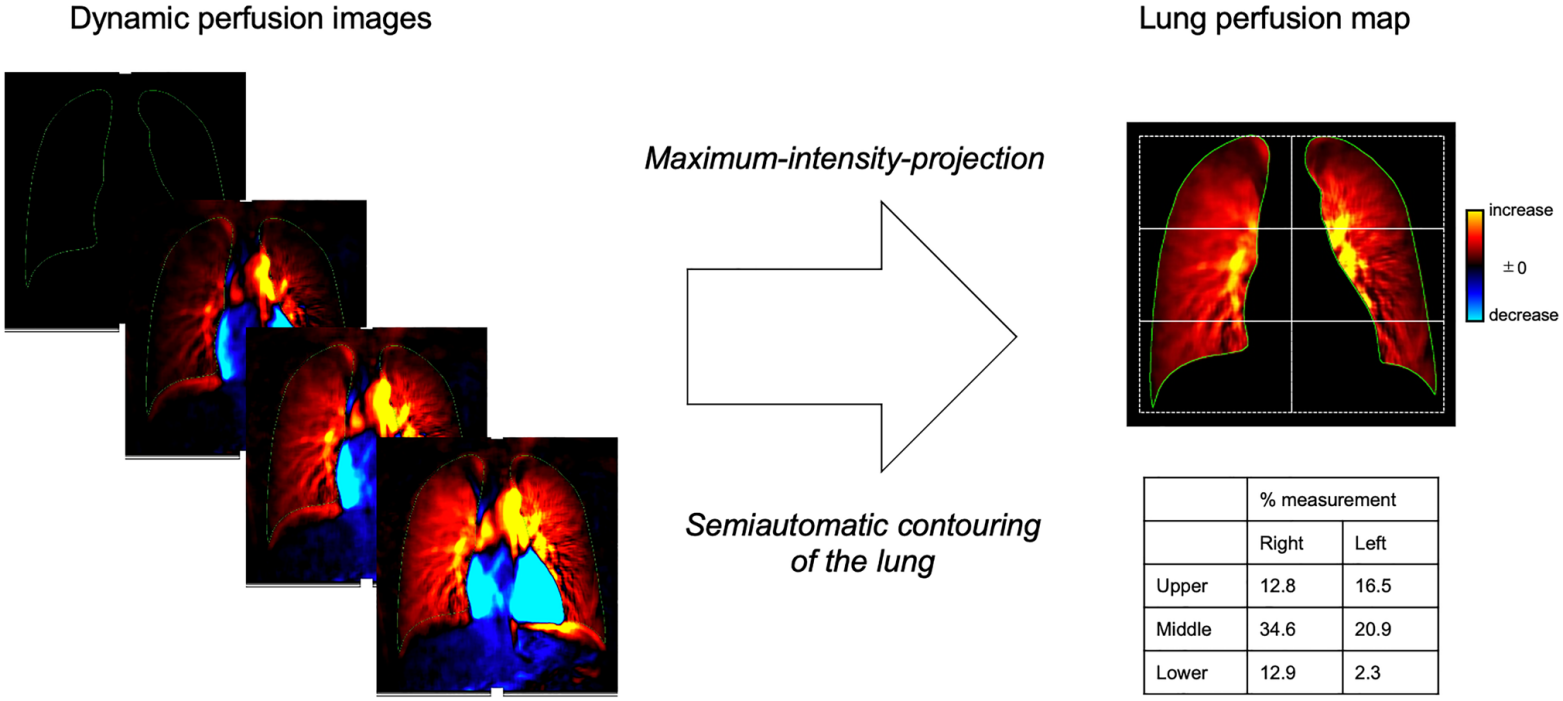
Creation of a lung perfusion map from dynamic perfusion images of dynamic chest radiography. The maximum intensity projection image in the lung, which is determined using semiautomatic contouring, is created as a lung perfusion map. Each lung is divided into three zones (upper, middle, and lower), and the percentage of measurements are calculated

This method is suitable for quantifying blood flow by representing the magnitude of the pulse waveform strength. Similar to perfusion scintigraphy, it can be used to semi-quantitatively evaluate regional pulmonary perfusion. The maximum intensity projection image of the lungs, which is determined by semiautomatic contouring, is created as a lung perfusion map. Each lung is divided into three zones (upper, middle, and lower) and the percentage of measurements are calculated (Fig. 6). DCR-derived perfusion metrics have been proven to correlate reasonably well with nuclear medicine imaging findings, suggesting that DCR can provide useful information on pulmonary function [5, 15–17]. This method holds significant promise in the field of cardiovascular analysis and provides valuable information for clinical decision making.

### Advantages compared to other imaging modalities

DCR has several advantages over other imaging modalities, resulting in many clinical applications.

First, because DCR is a non-invasive method that does not require the use of contrast media or radionuclides, it is associated with almost no contraindications. Furthermore, it is a rapid and readily available imaging technique, requiring only 7–10 s for capture and 1 min for post-processing analysis. This facilitates the diagnostic process in emergency medicine and strengthens the diagnostic power.

The installation cost of DCR is lower than that of CT, magnetic resonance imaging (MRI), single-photon emission computed tomography, and interventional angiography systems. Moreover, additional costs, such as radioisotopes or contrast materials for each use, are not incurred.

The entrance surface doses for DCR can be less than the dose limit for the two projections (posteroanterior and lateral views) of chest radiographs recommended by the International Atomic Energy Agency [18, 19]. Compared with other imaging modalities, the radiation dose of perfusion DCR is one-tenth that of lung ventilation/perfusion (V/Q) scanning and one-twentieth that of standard CTPA. The doses for DCR, V/Q scan, and CTPA are approximately 0.2, 2, and 4–6 mSv, respectively [6].

Several other imaging modalities can be used to evaluate pulmonary perfusion such as CTPA, perfusion scintigraphy, and MR angiography. CTPA is rapid and readily available; however, high radiation exposure and the need for contrast media may cause problems in patients with allergies to contrast media, impaired renal function, or radiosensitivity. V/Q scintigraphy, which has almost no contraindications, is a good alternative for such patients, but is not readily available because of its high specialty and large equipment. The use of MRI is heavily dependent on local practice and is routinely implemented in only a few high-volume institutions with clustered expertise. In addition, MRI is technically challenging, expensive, and has limited availability. DCR could be an effective alternative in conditions that limit the use of these imaging modalities (e.g., local hospitals that do not have large imaging equipment, or patients with contrast allergies who attend emergency departments).

### Three steps on how to interpret DCR perfusion images

To assess DCR, a comparison between chest radiographs and perfusion images is necessary.

**Fig. 7:**
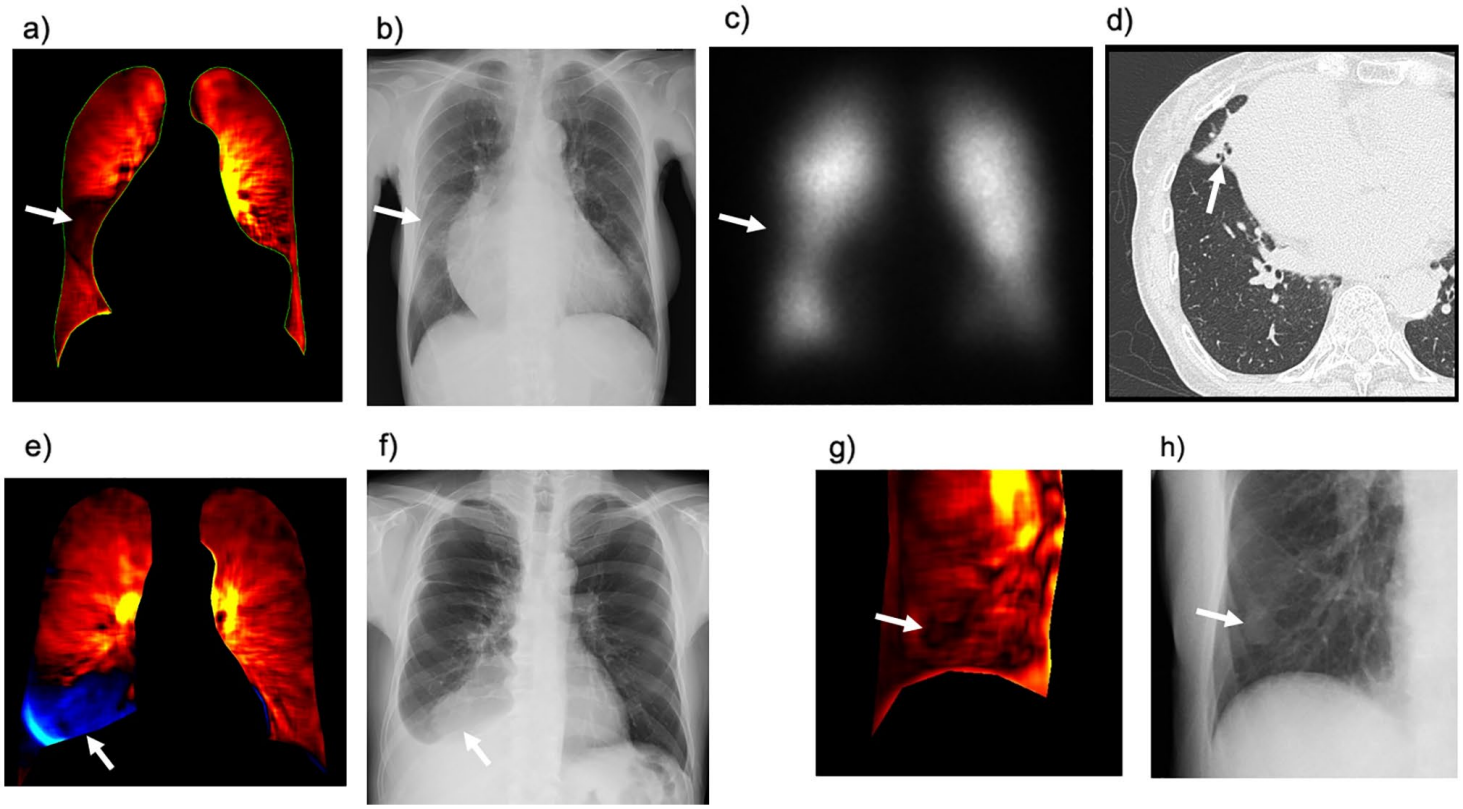
Typical pseudo-lesions of perfusion images of dynamic chest radiography (DCR): signal defects due to pulmonary or extrapulmonary lesions. Perfusion images of DCR (**a**), chest radiograph (**b**), anterior planar perfusion scintigraphy (**c**), and chest computed tomography (CT) (**d**) in a patient with atelectasis of the right middle lobe. DCR perfusion image (**e**) and chest radiograph (**f**) of a patient with right pleural effusion. DCR perfusion image (**g**) and chest radiograph (**h**) of the nipple shadow

First, we read sequential DCR images or chest radiographs to assess lung abnormalities (mass/nodule, opacity, reticulation, bulla, pneumothorax, and pleural effusion) and reference the lung border. Subsequently, we read both the dynamic perfusion images of the DCR and the lung perfusion map to detect perfusion abnormalities. Finally, both the images are compared. The findings are considered abnormal when the abnormality in the dynamic perfusion images or lung perfusion map is accompanied by normal lung findings in the corresponding lung area in the sequential images of DCR or chest radiography. This process effectively avoids the overdiagnosis of pulmonary perfusion abnormalities due to nonvascular lesions. Typical pseudo-lesions and signal defects due to pulmonary or extrapulmonary lesions are shown in Fig. 7.

### Clinical applications and correlation with other imaging modalities

#### Acute PE

**Fig. 8:**
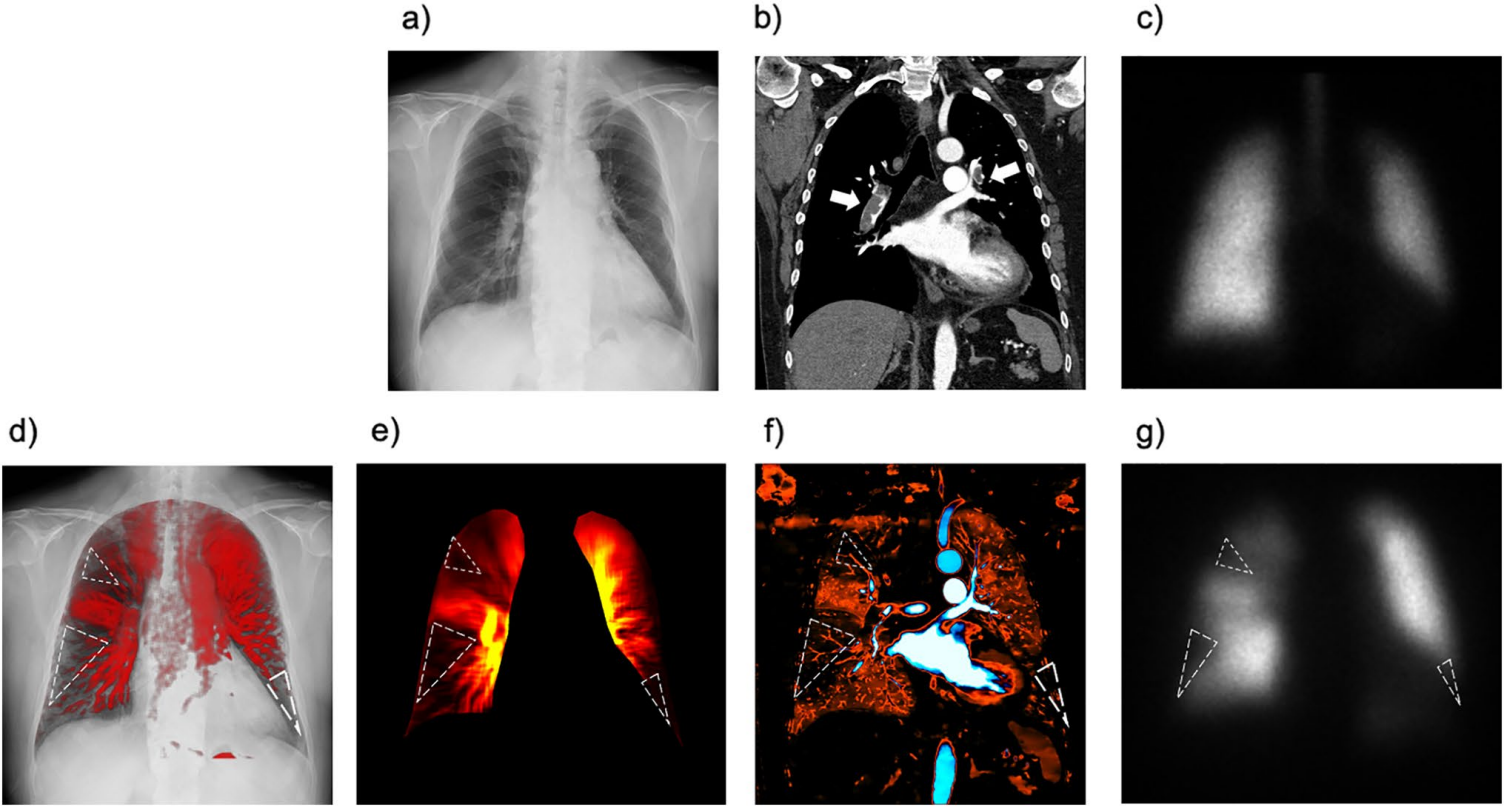
Images of a 50-year-old woman with acute pulmonary embolism. Normal findings are shown in the chest radiograph (**a**) and anterior planar ventilation scintigraphy (**c**), whereas the coronal view of the CT pulmonary angiography shows large blood clots in the bilateral pulmonary arteries (**b**, arrows). Multiple wedge-shaped perfusion defects (dotted triangles) are demonstrated in the bilateral lungs in perfusion images obtained using the cross-correlation method (**d**) and reference frame subtraction method (**e**) of dynamic chest radiography, an iodine map created from computed tomography pulmonary angiography (**f**), and anterior planar perfusion scintigraphy (**g**). *Reproduced with permission from Ref 16 (Yamasaki Y, et al. Dynamic Chest Radiography of Acute Pulmonary Thromboembolism. Radiology: Cardiothoracic Imaging 2022; 4(4):220,086)

Acute PE is a sudden blockage of the pulmonary arteries by blood clots, which leads to reduced lung perfusion and lung tissue injury. Acute PE is relatively common, with an annual incidence of 39–115 per 100 000 population [20], and is a potentially life-threatening condition with an age-adjusted mortality rate (PE-related death) of 14.4% [21]; therefore, a readily available diagnostic method without contraindication is preferable. DCR typically shows triangular or wedge-shaped defects similar to perfusion scintigraphy and the iodine map created from CTPA in acute PE (Fig. 8 and Video 2) [22]. Owing to its high diagnostic accuracy, CTPA is a highly recommended non-invasive modality for patients with suspected PE [20]. However, the use of contrast media may occasionally be problematic in patients with contrast allergies or impaired renal function. Although V/Q scintigraphy is a suitable alternative for such patients, it is not always available at most institutes or hospitals [23]. DCR should be considered in such a scenario and may be helpful. However, the sensitivity of DCR for acute PE is lower than that of CTPA, and a comprehensive approach combined with D-dimer testing and clinical pre-test probability, which can be performed even in clinical situations where CTPA is unavailable, is preferable [24, 25].

According to a systematic review, silent PE occurs in approximately one-third of the patients with deep venous thrombosis [26]. Moreover, silent PE may occur in the central pulmonary arteries. Therefore, DCR may be an effective tool for screening silent and large PEs in patients with DVT.

#### Chronic thromboembolic pulmonary hypertension (CTEPH)

**Fig. 9:**
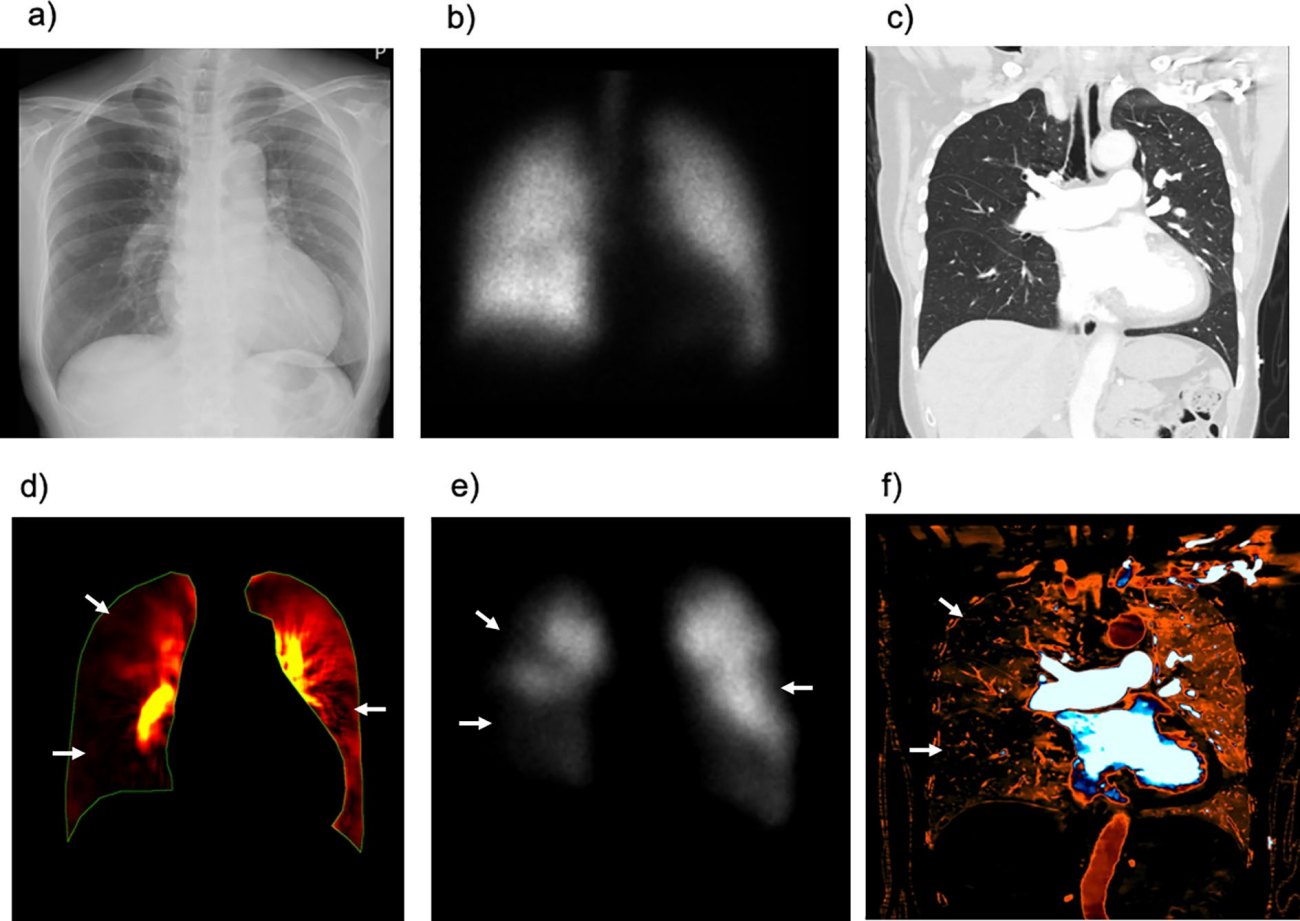
Images of a 67-year-old woman with chronic thromboembolic pulmonary hypertension. Normal lung findings are shown in the chest radiograph (**a**), anterior planar ventilation scintigraphy (**b**), and coronal view of the chest computed tomography (CT) (**c**), whereas wedge-shaped perfusion abnormalities are observed in the perfusion image on dynamic chest radiography (**d**), anterior planar perfusion scintigraphy (**e**), and iodine map created from CT pulmonary angiography (**f**)

**Fig. 10:**
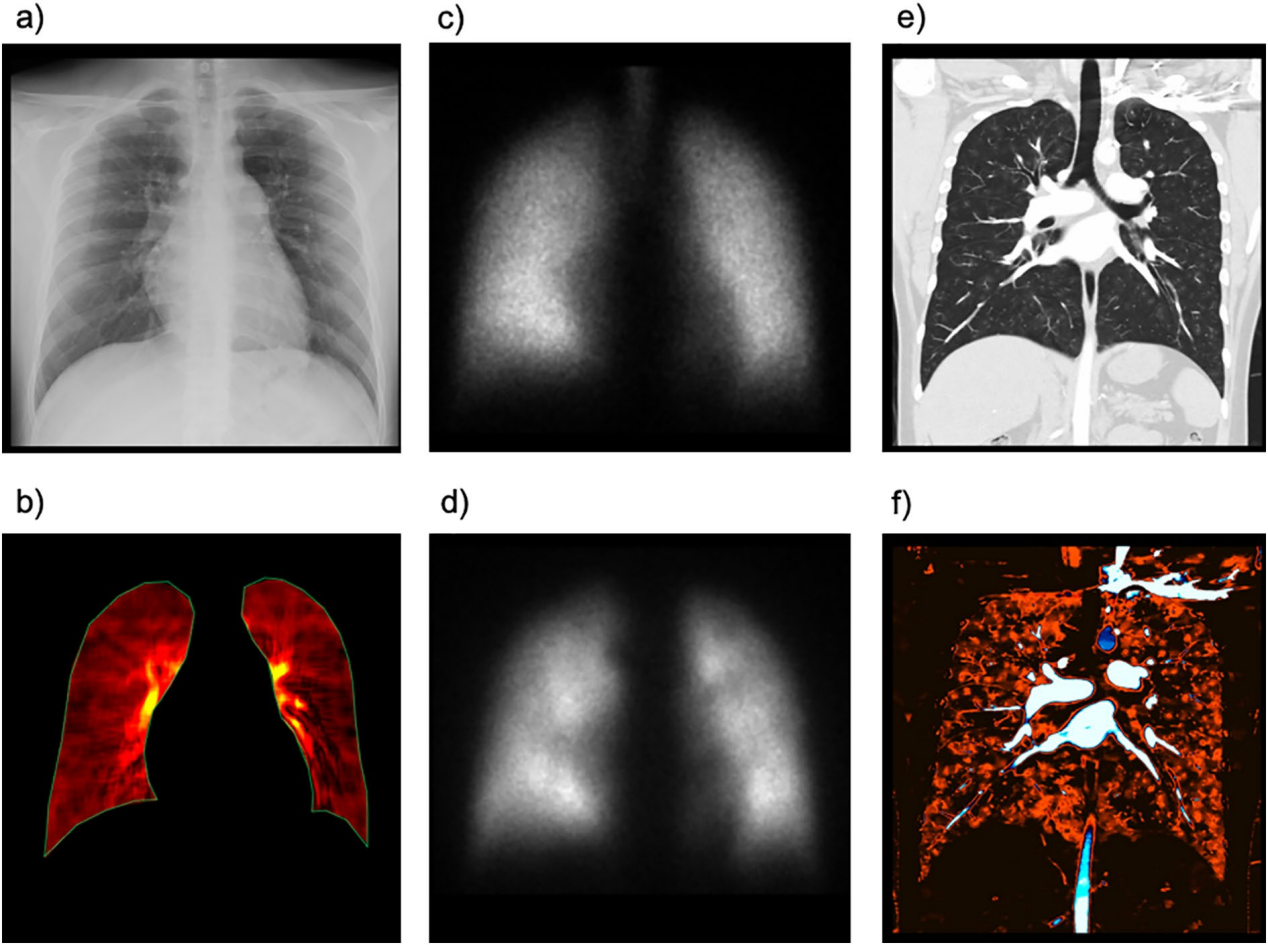
Images of a 21-year-old man with familial pulmonary arterial hypertension. Normal lung findings are shown on the chest radiograph (**a**) and perfusion image of the dynamic chest radiograph (**b**). No definite ventilation/perfusion mismatch is observed on anterior planar ventilation/perfusion scintigraphy (**c**, **d**). Chest computed tomography (CT) showing multiple subtle centrilobular ground-glass opacities in both lungs (**e**). Iodine images derived from CT pulmonary angiography showing slightly heterogeneous enhancement corresponding to lung opacities; however, no embolic-type defect was observed in either lung (**f**)

PH is divided into five groups: pulmonary arterial hypertension (Group 1), PH due to left heart disease (Group 2), PH due to lung diseases and/or hypoxia (Group 3), PH due to pulmonary artery obstructions including CTEPH (Group 4), and PH with unclear and/or multifactorial mechanisms (Group 5). CTEPH is a subtype of PH that occurs because of unresolved pulmonary thromboembolism and causes persistent obstruction of pulmonary vessels, progressive pulmonary artery remodeling, and PH [27]. If left untreated, patient prognosis is poor due to subsequent right heart failure and death [28, 29]. Pulmonary endarterectomy, pulmonary vasodilation, and balloon pulmonary angioplasty improve pulmonary hemodynamics and exercise tolerance in patients with CTEPH [30]. Delayed diagnosis negatively affects the prognosis of CTEPH; thus, early detection and treatment are essential for improving clinical outcomes [28, 31, 32]. Current guidelines require a V/Q lung scan as the first step in diagnosing chronic PE in PH because of its high diagnostic accuracy [33]. However, it is underused for the diagnosis of CTEPH worldwide [34]. A method with greater availability and less radiation exposure for detecting CTEPH is clinically desirable to enable early detection and treatment, resulting in an improved prognosis. Similar to perfusion scintigraphy and invasive pulmonary angiography, DCR shows triangular or wedge-shaped defects (Fig. 9) [35]. Notably, CTEPH differs from other etiological PHs on DCR, as does perfusion scintigraphy (Fig. 10) [36]. In a small pilot study, DCR showed an efficacy similar to that of a V/Q scan for the detection of CTEPH [6]. DCR may be an effective alternative to V/Q scanning for the diagnosis of CTEPH; however, a prospective validation study with a larger sample size is required.

**Fig. 11:**
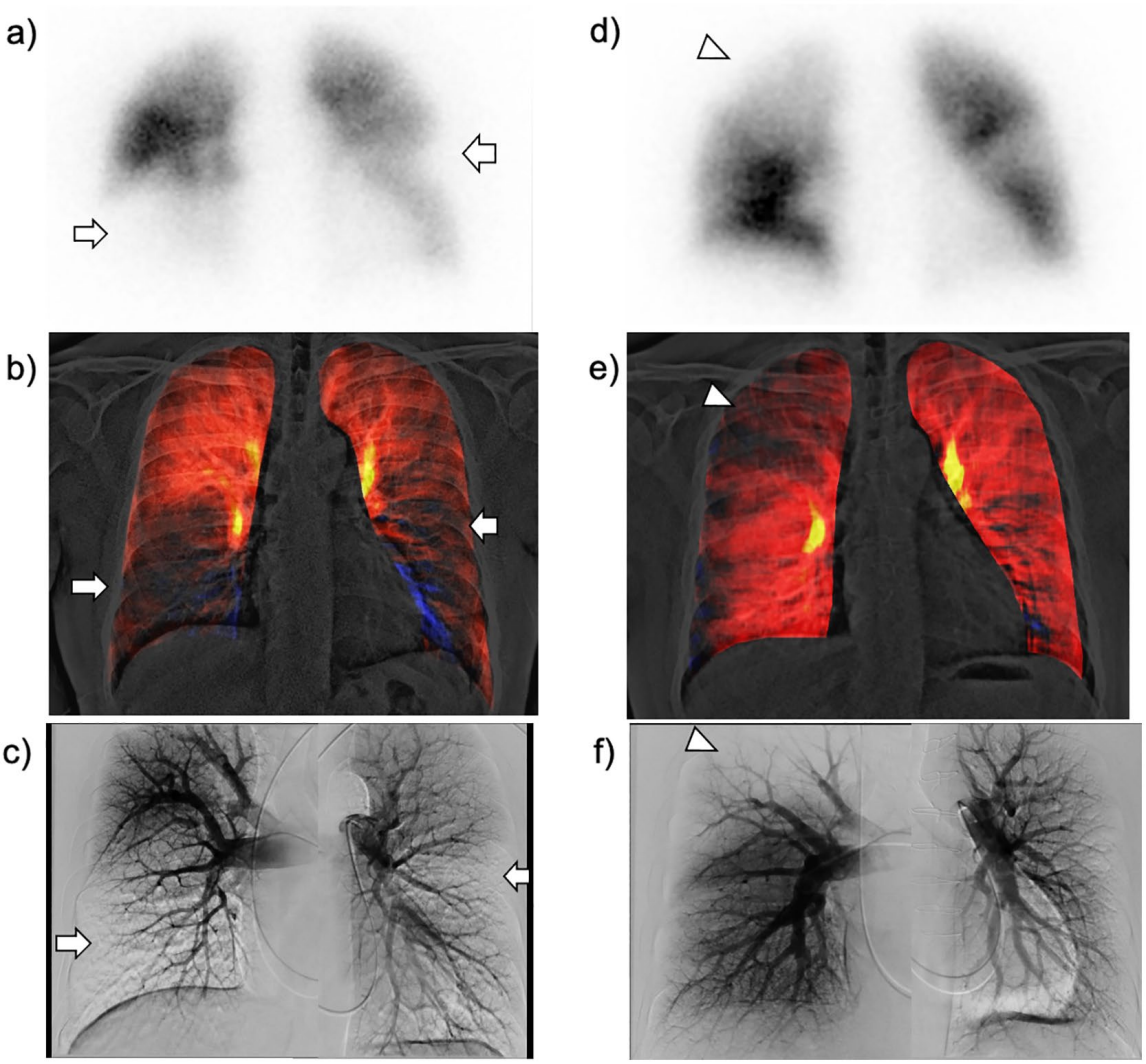
A 46-year-old man with chronic thromboembolic pulmonary hypertension before (**a**–**c**) and after (**d**–**f**) pulmonary endarterectomy (PEA). Tc-99 m macroaggregated albumin lung perfusion scintigraphy showing multiple perfusion defects in both the lungs (a, arrows). A lung perfusion image created from dynamic chest radiography (DCR) demonstrates lung perfusion defects (**b**), similar to lung perfusion scintigraphy and pulmonary angiography (**a**, **c**). After PEA, the lung perfusion image of DCR (**e**) demonstrates improvement in lung perfusion in the lower lungs, bilaterally, with a relative decrease in the right upper lung, which is the so-called vascular steal (**d**–**f**, arrowheads). *Reproduced with permission from Ref 27 (Yamasaki Y, et al. A novel pulmonary circulation imaging using dynamic digital radiography for chronic thromboembolic pulmonary hypertension. Eur Heart J. 2020;41(26):2506)

**Fig. 12:**
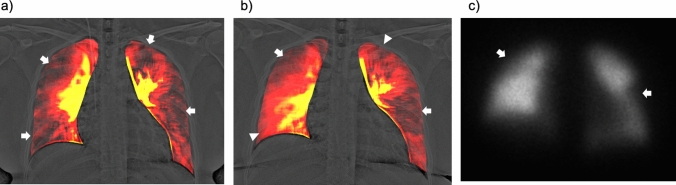
Dynamic chest radiography (DCR)-based detection of acute pulmonary thromboembolism (PTE) progression to chronic thromboembolic pulmonary hypertension (CTEPH) in a 32-year-old woman. In the acute PTE phase, DCR demonstrated multiple perfusion defects, suggesting pulmonary embolism in both lungs (**a**, arrows). Even after 6 months of anticoagulation therapy, the patient experienced mild dyspnea. Repeat DCR demonstrates improved perfusion defects (**b**, arrowheads) and persistent large perfusion defects in the bilateral lung fields (**b**, arrows), which are very similar to the findings of subsequent ventilation/perfusion (V/Q) scintigraphy (V/Q mismatch; **c**, arrows). Pulmonary hypertension was confirmed using invasive right heart catheterization, and a diagnosis of CTEPH was confirmed. *Reproduced from Ref 29 (Yamasaki Y, et al. Chronic thromboembolic pulmonary hypertension after acute pulmonary thromboembolism revealed by dynamic chest radiography. Eur Heart J Cardiovasc Imaging. 2022;23(6):e264-e265), which is an open-access article distributed under the Creative Commons Attribution License

Additionally, DCR demonstrates improvement and shift changes in lung perfusion after pulmonary endarterectomy in patients with CTEPH (Fig. 11 and Video 3) [35]. Similar to perfusion scintigraphy, DCR demonstrates residual pulmonary perfusion defects in patients with CTEPH that develop after acute PE (Fig. 12 and Video 4) [37]. CTEPH is a serious complication of acute PE. Approximately, 3.4% of the survivors of acute PE develop CTEPH [38]. The advantages of DCR, such as its non-invasiveness, easy availability, and low radiation exposure, would be helpful for repeat assessments of pulmonary perfusion during clinical follow-up.

#### Pitfalls in diagnosing PE using DCR

**Fig. 13:**
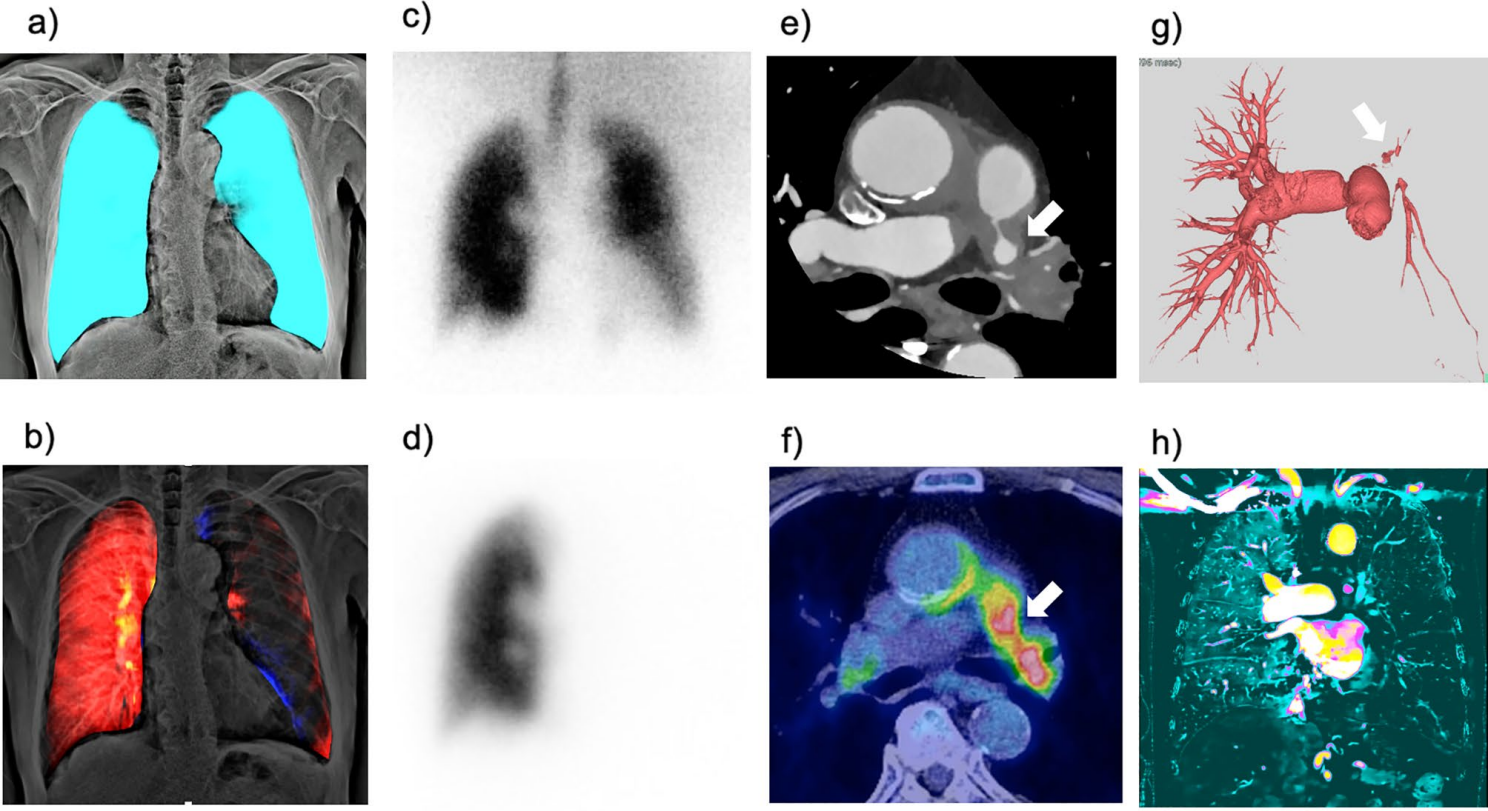
Images of a 74-year-old man with giant cell arteritis. An ipsilateral perfusion defect with normal ventilation (ventilation/perfusion mismatch) in the left lung is observed on ventilation (**a**) and perfusion (**b**) images of dynamic chest radiography, similar to the findings of ventilation (**c**)/perfusion (**d**) scintigraphy. Computed tomography pulmonary angiography (CTPA) and 18F-fluorodeoxyglucose positron emission tomography (FDG-PET) revealed severe stenosis of the left pulmonary artery due to pulmonary arterial wall thickening (e, g, arrows) with high FDG uptake (maximum standardized uptake value, 7.6) (f, arrow), indicating large-vessel vasculitis. The iodine map created from CTPA also shows a finding similar to that of the perfusion image from dynamic chest radiography (h). *Reproduced with permission from Ref 31 (Yamasaki Y et al. Pulmonary ventilation–perfusion mismatch demonstrated by dynamic chest radiography in giant cell arteritis. Eur Heart J. 2021;42(2):208–209)

Acute and chronic PEs are major targets for the clinical application of DCR. Triangular or wedge-shaped perfusion defects are typical findings of PE on DCR and originate from segmental or lobar perfusion abnormalities; however, several causes of vascular occlusion/stenosis can also cause lobar/segmental perfusion defects. Other vascular causes include vascular compression/compromise from the tumor or fibrosing mediastinitis, vasculitis affecting the pulmonary vessels (Fig. 13 and Video 5) [39], altered pulmonary circulation due to pulmonary artery hypoplasia or pulmonary sequestration, and granulomatous disease affecting the vessels, such as pulmonary sarcoidosis.

Moreover, it is difficult to detect small perfusion abnormalities, such as small PE, which are unclear in the anterior view of planar perfusion scintigraphy.

#### PH

**Fig. 14:**
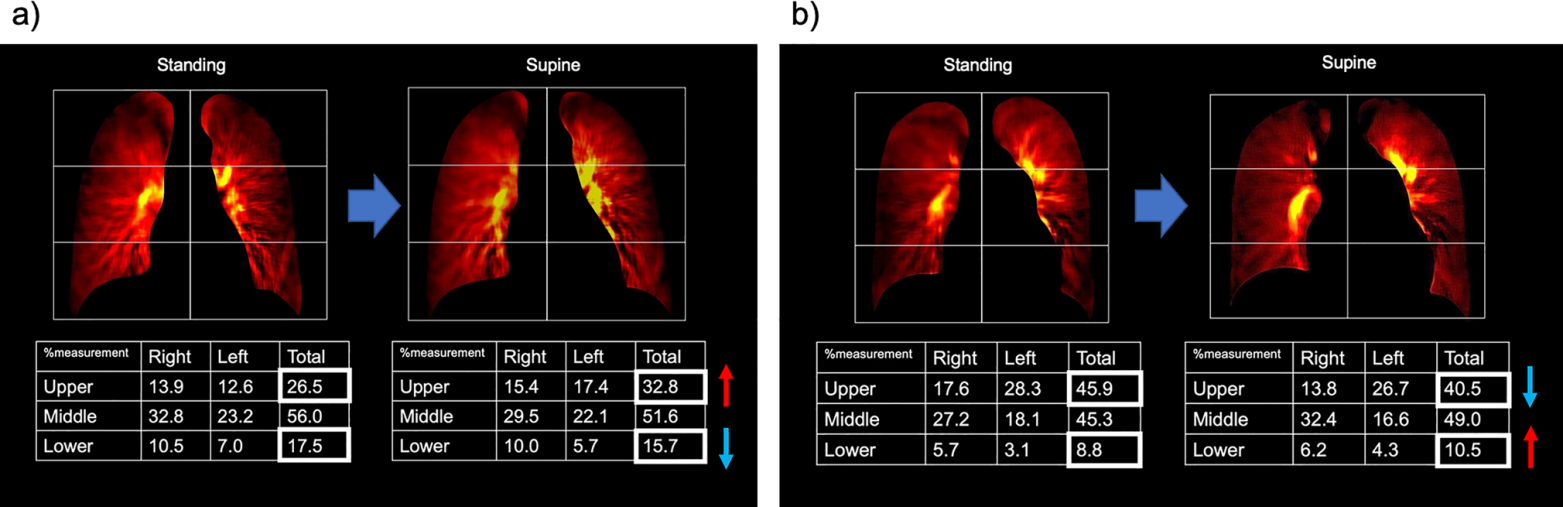
Postural changes in pulmonary perfusion assessed by the perfusion map of dynamic chest radiography in a healthy volunteer (**a**) and a patient with pulmonary arterial hypertension (**b**). With the postural change from the standing to supine position, the percentage measurement of the upper lung increased (from 26.5% to 32.8%) and that of the lower lung decreased (from 17.5% to 15.7%) in a healthy volunteer (**a**), whereas the percentage measurement of the upper lung decreased (from 45.9% to 40.5%) and that of the lower lung increased (from 8.8% to 10.5%) in a patient with pulmonary arterial hypertension (**b**)

**Fig. 15:**
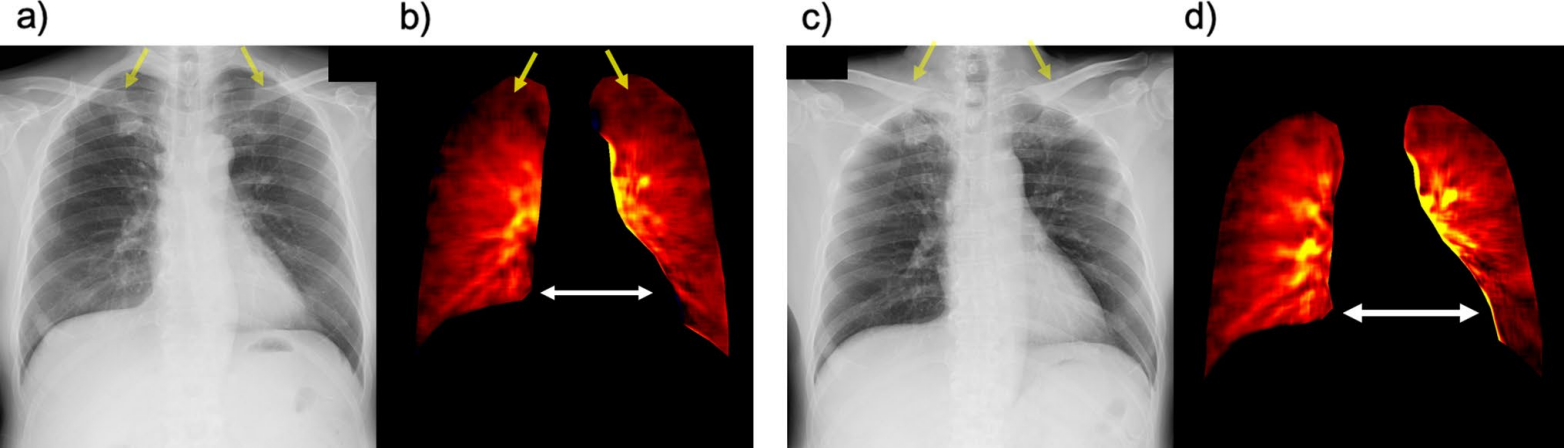
Images of a 66-year-old man without lung diseases. Clavicles superimposed on bilateral upper lungs on the chest radiograph captured in standing position (**a**, arrows), leading to a slight decrease in pulmonary perfusion in the dynamic perfusion image (**b**, arrows). In contrast, the clavicles shift upward in the chest radiograph captured in the supine position (**c**, arrows) so that pulmonary perfusion in the upper lungs is clearly visible (**d**). Furthermore, the heart shadow in the dynamic perfusion image captured in the supine position (**d**, two-headed arrows) is larger than that captured in the standing position (**b**, two-headed arrows), which may have led to decreased lung perfusion in the left lower lung

DCR can be used to clinically evaluate postural changes of pulmonary perfusion. Gravity-dependent perfusion redistribution relies on a low pulmonary arterial pressure and the ability to distend and/or recruit previously underperfused blood vessels. However, as the pulmonary arterial pressure increases, the influence of gravity on regional lung perfusion proportionally loses significance [10, 40]. A study reported that DCR could detect postural changes in pulmonary blood flow using a monkey model [3]. DCR may detect differences in postural changes in pulmonary perfusion in patients with PH (Fig. 14). However, the standing scan is performed in the posteroanterior (PA) direction, while the supine scan is performed in the anteroposterior (AP) direction. This difference may influence the interpretation of the results. Specifically, the AP projection produces a slightly wider mediastinal shadow than the PA projection because of the increased distance of the heart from the image receptor, resulting in an underestimation of pulmonary perfusion in the lower lungs. In contrast, the clavicles, which might obscure the change in pixel values in the lungs, tend to shift upward in the AP view compared to in the PA view, thereby avoiding the underestimation of pulmonary perfusion in the upper lungs (Fig. 15).

#### ACHD

**Fig. 16:**
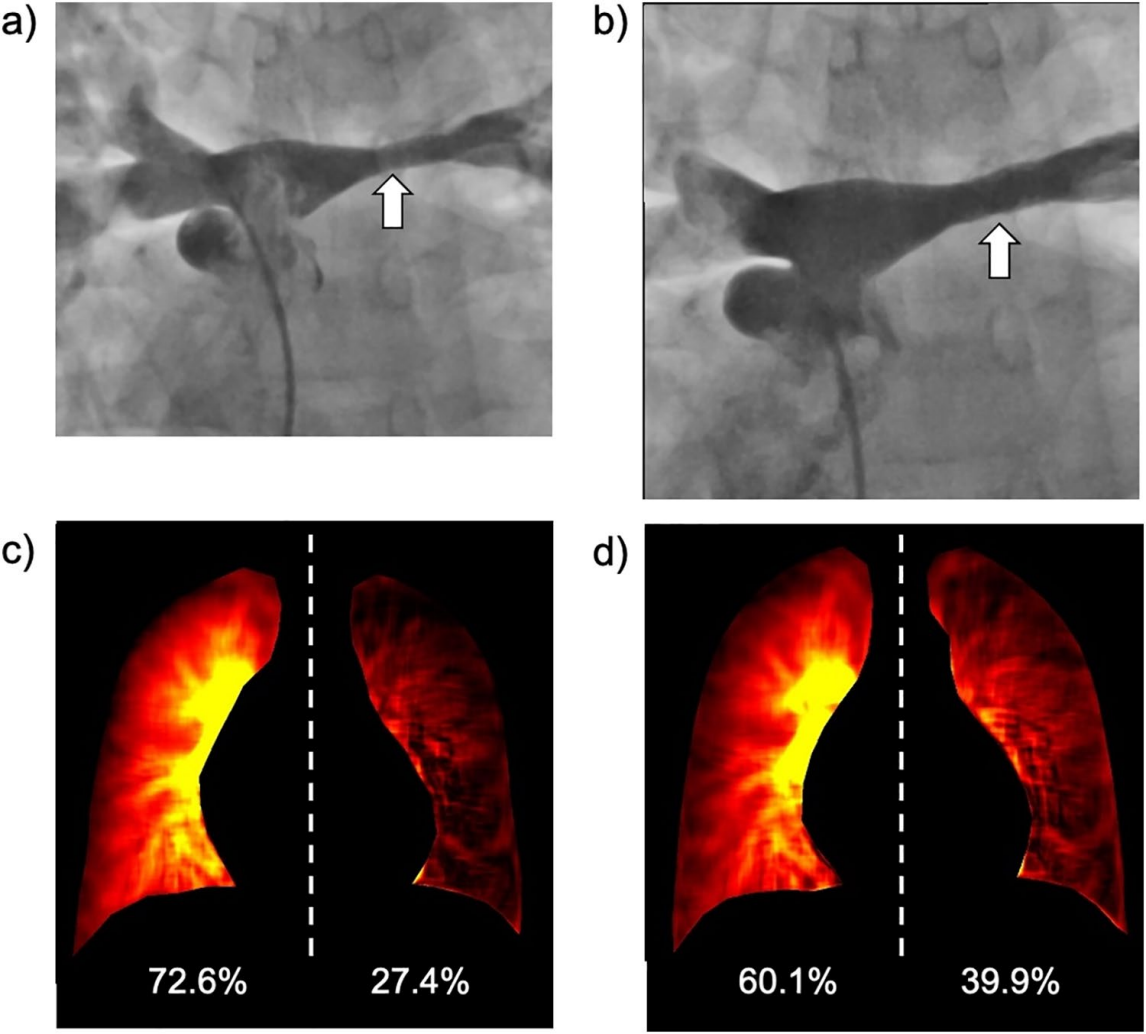
Images of a 17-year-old boy with repaired transposition of the great arteries before and after stent placement for the left pulmonary artery stenosis. The pressure gradient between the main and left pulmonary arteries was 13 mmHg before and 8 mmHg after treatment. Pulmonary angiography showing left pulmonary artery stenosis (**a**, arrow) and improvement after stent placement (**b**, arrow). Semi-quantitative evaluation of pulmonary perfusion using the perfusion map of dynamic chest radiography (**c**) showed similar values (right 72.6%, left 27.4%) to those of phase-contrast magnetic resonance imaging (76.0%, 24.0%) and perfusion scintigraphy (70.4%, 29.6%). The improvement in maldistribution after treatment is also demonstrated (60.1%, 39.9%) (**d**)

Patients with ACHD occasionally develop pulmonary artery stenosis. Maldistribution of the pulmonary artery flow is usually evaluated using perfusion scintigraphy or phase-contrast MRI. However, they could be associated with challenges, such as inability to remain still and repeat radiation exposure, because patients with ACHD are mostly young and sometimes have mental disorders [41]. DCR demonstrates findings similar to perfusion scintigraphy and invasive pulmonary angiography with a short examination time and facilitates both qualitative and quantitative evaluations of pulmonary circulation (Fig. 16) [42]. This convenient and non-invasive method is expected to be an effective alternative approach for assessing lung perfusion in patients with pulmonary artery stenosis.

#### Pulmonary arteriovenous malformation (AVM)

**Fig. 17:**
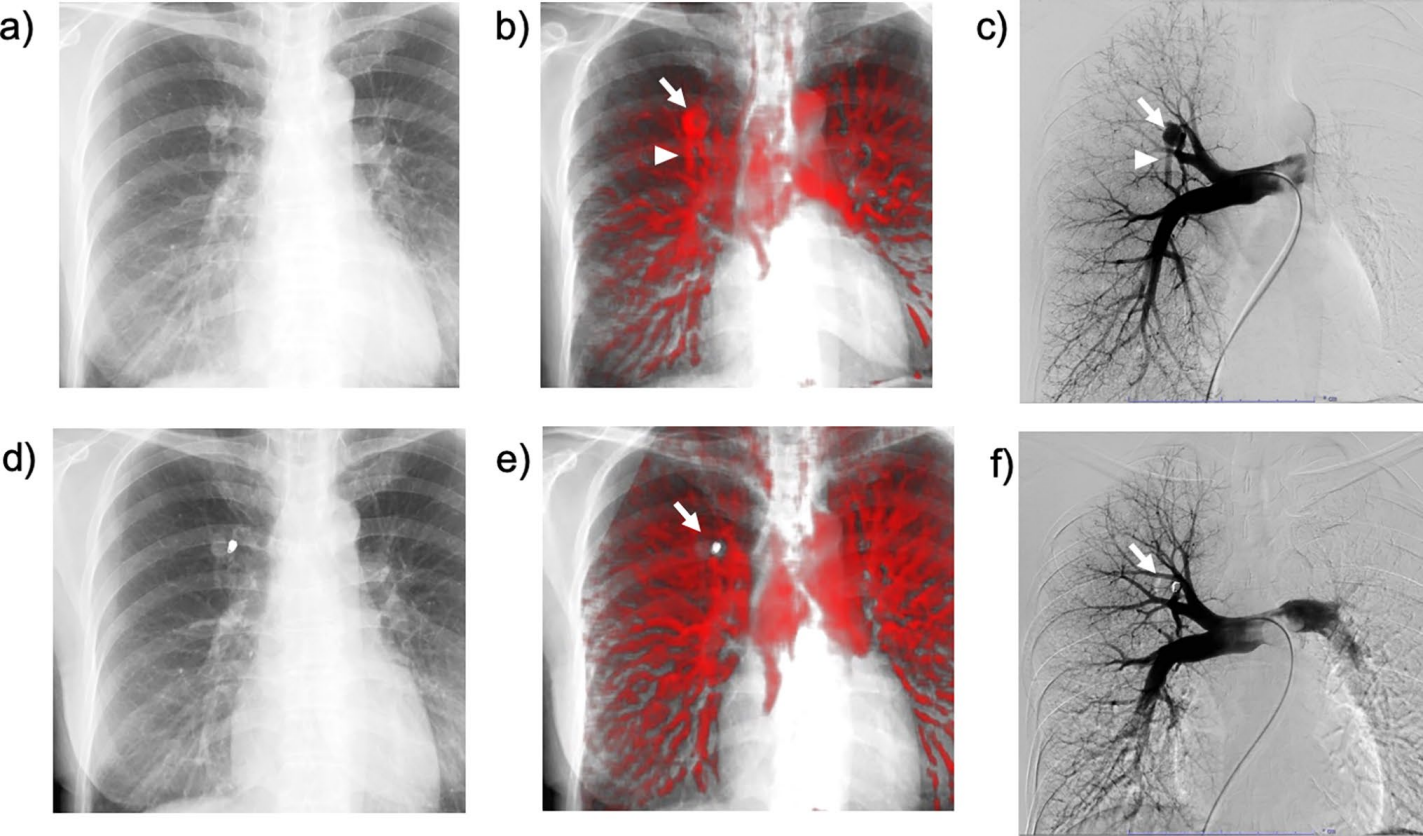
Images of a 52-year-old woman with a simple pulmonary arteriovenous malformation (one feeding artery and one drainage vein) before and after coil embolization of the feeding artery. The chest radiograph (**a**) shows a solitary nodule in the right upper lung. Perfusion image of the cross-correlation method of dynamic chest radiography (DCR) (**b**) shows enhancement of the nidus (arrow) and drainage vein (arrowhead), similar to invasive angiography (**c**, arrow, and arrowhead). After coil embolization of the feeding artery, a chest radiograph shows slightly reduced opacity of the lung nodule (**d**). DCR perfusion images demonstrate loss of enhancement (**e**, arrows), similar to invasive angiography (f, arrows). *Reproduced with permission from Ref 7 (Yamasaki Y and Ishigami K. Dynamic chest Radiography of Pulmonary Arteriovenous Malformation. Radiology. 2021;300(2):285)

A pulmonary AVM is a fistulous connection between the pulmonary artery and vein through the nidus or sac, bypassing the normal pulmonary capillary bed, and resulting in a right-to-left shunt. The symptoms include dyspnea, hemoptysis, cerebral stroke, and brain abscesses. Its prevalence is 1/2500; 80% of the cases are associated with hereditary hemorrhagic telangiectasia [43, 44]. Similar to invasive angiography, DCR shows enhancement of the nidus (Fig. 17 and Video 6); further, loss of enhancement after coil embolization of the feeding artery has been demonstrated [7].

**Fig. 18:**
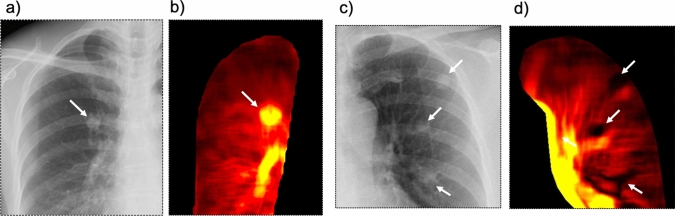
Images of patients with pulmonary arteriovenous malformations (AVM) (**a**, **b**) and pulmonary metastases of cervical cancer (squamous cell carcinoma) (**c**, **d**). Chest radiography illustrating similar well-circumscribed lung nodules in both patients (**a**, **c**, arrows), although a linear shadow connected to the nodule, which indicated feeding arteries or drainage veins, is observed in one patient with pulmonary AVM. In contrast, a markedly increased signal is observed in a lung nodule in a pulmonary AVM (**b**, arrow), whereas a low signal is observed in lung nodules in lung metastases (**d**, arrow)

Interestingly, a markedly increased signal is observed in the pulmonary AVM, whereas a low signal is observed in the lung carcinomas and metastases (Fig. 18). These findings may be useful for differentiating between vascular lesions and tumors.

#### Pulmonary vein stenosis (PVS)

**Fig. 19:**
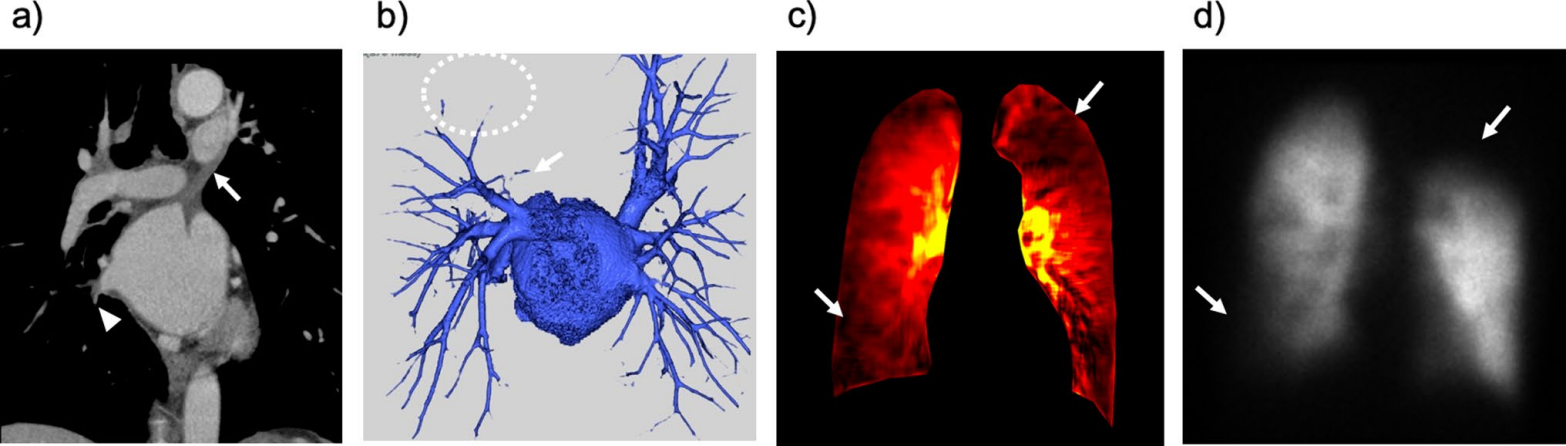
Images of a 62-year-old man with pulmonary vein stenosis following catheter ablation for atrial fibrillation. Occlusion of the left upper pulmonary vein (arrows, Fig. **a** and **b**), severe stenosis of the right lower pulmonary vein (arrowhead, Fig. **a**), and an avascular area in the left upper lung (dotted circle, Fig. **b**) were observed in the coronal view of contrast-enhanced computed tomography (CT) and a posterior view of the 3D image of the pulmonary vein created by contrast-enhanced CT. Hypoperfusion in the right lower and left upper lungs is illustrated in the perfusion image of dynamic chest radiography (arrows, Fig. **c**), similar to anterior planar perfusion scintigraphy (arrows, Fig. **d**)

Severe PVS is a rare, but life-threatening complication of catheter-based ablation in patients with atrial fibrillation. The incidence of severe PVS is 0.32–3.4% [45]. Symptoms are nonspecific (e.g., dyspnea, cough, fatigue, and decreased exercise tolerance); therefore, PVS is frequently misdiagnosed as pneumonia or other diseases [45–47]. Diagnostic modalities include contrast-enhanced CT, V/Q scan, and invasive angiography. However, the diagnosis is often delayed because routine imaging after ablation is not mandated by the current Heart Rhythm Society consensus statement [48]. Similar to perfusion scintigraphy, DCR demonstrates decreased perfusion in the lung zone corresponding to the PVS (Fig. 19). The easy availability and non-invasiveness of DCR may make it suitable for routine imaging after ablation and contribute to the early detection of PVS.

#### Partial anomalous pulmonary venous return (PAPVR)

**Fig. 20:**
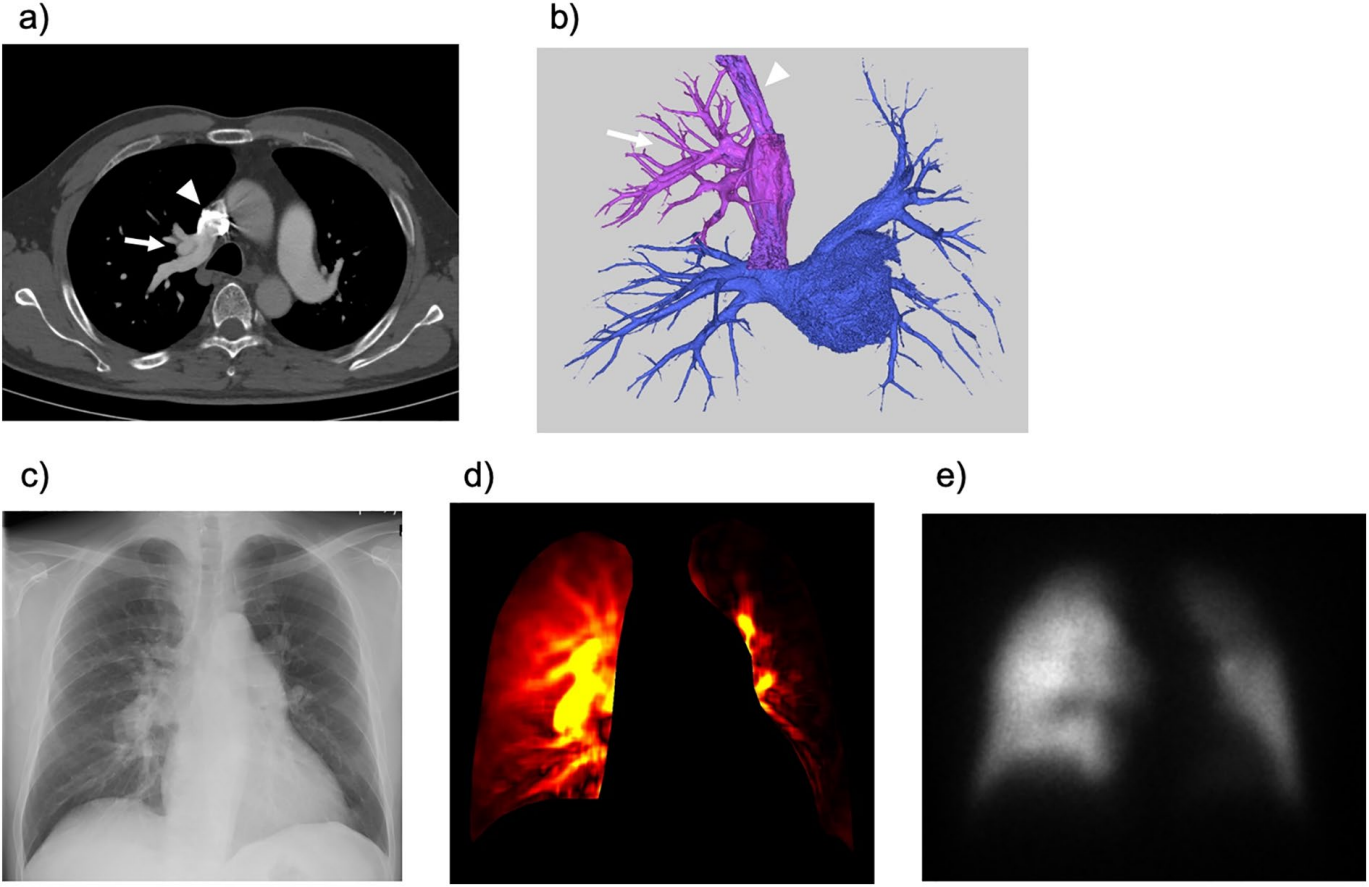
Images of a 50-year-old man with partial anomalous pulmonary venous return (PAPVR). Axial image (**a**) and 3D image of the pulmonary vein (**b**) obtained from computed tomography pulmonary angiography showing the right upper pulmonary vein (arrows) draining into the superior vena cava (arrowheads). Chest radiograph showing prominent pulmonary vasculature in the right lung (**c**). Dynamic chest radiography (**d**) shows increased pulmonary perfusion in the right lung (right, 85.6%; left, 14.4%), similar to perfusion planar scintigraphy (right 73.8%, left 26.8%) (**e**)

PAPVR is a congenital abnormality in which some, but not all, pulmonary veins connect to the right atrium or one of its venous tributaries. PAPVR is a cause of adult-onset pulmonary arterial hypertension that is often overlooked [49, 50]. Similar to perfusion scintigraphy, DCR demonstrates increased pulmonary perfusion in lungs with PAPVR (Fig. 20). Adding DCR to conventional chest radiographic screening may help detect PAPVR.

### Limitations

DCR has several limitations. First, the left lower lung is prone to motion artifacts due to heartbeats. The percentage measurement in the left lower lung is expected to be underestimated in DCR, owing to the shadow of the heart and susceptibility to motion artifacts from heartbeats. Therefore, DCR has a limitation in the evaluation of the left lower lung. Second, the patient’s inability to remain still or hold breath for 7–10 s could produce motion artifacts. Finally, whether 7–10 s of X-ray irradiation from a DCR interferes with pacemakers and implantable cardioverter defibrillators and causes clinically significant adverse events remains undetermined. Currently, DCR should be avoided in patients using these devices.

### Future perspective

Long coronavirus disease (COVID) is a debilitating illness that occurs in at least 10% of severe acute respiratory syndrome coronavirus 2 infections. Respiratory conditions are a common phenotype in long COVID, and a study reported that they occurred twice as often in COVID-19 survivors than in the general population [51]. Shortness of breath and cough are the most common respiratory symptoms and persist for at least 7 months in 40% and 20% of patients with long COVID, respectively [52]. Several imaging studies that included nonhospitalized individuals with long COVID demonstrated lung perfusion abnormalities [53]; thus, examining lung perfusion in such patients is important. The repeatability of DCR, owing to its non-invasiveness, lack of need of contrast, and low radiation, make it suitable for follow-up assessment.

When disasters such as earthquakes occur, the prevalence of deep venous thrombi and PE increases [54, 55]. In addition, disasters occasionally disturb the prompt access to hospitals and medical supplies. DCR requires only a pulsed X-ray generator, an FPD, and analysis software, which have the potential to be installed in medical vehicles. The portability and accessibility of DCR are promising in disaster medicine.

PE that is suspected during pregnancy is a serious problem. Since the use of contrast medium and high radiation exposure should be avoided during pregnancy, DCR, which has low radiation and does not require contrast medium, may be beneficial in such situations.

## Conclusions

DCR is a novel imaging technique that demonstrates pulmonary perfusion, with almost no contraindications. To assess DCR, a comparison between perfusion images and chest radiographs is necessary. Imaging findings of DCR highly correlated with those of the anterior image of planar perfusion scintigraphy, the coronal view of the iodine map of CTPA, and invasive pulmonary angiography. DCR can evaluate many pulmonary vascular diseases visually and semi-quantitatively and has huge potential in clinical applications.

### Supplementary Information

Below is the link to the electronic supplementary material.

Supplementary file1 (MP4 9451 KB) Video 1. Movie of dynamic perfusion images obtained using the cross-correlation analysis method in a 31-year-old healthy man (a) and the reference frame subtraction analysis method in a 39-year-old healthy man (b).

Supplementary file2 (MP4 3671 KB) Video 2. Movie of dynamic perfusion images obtained using the cross-correlation analysis method (a) and reference frame subtraction analysis method (b) in a 50-year-old woman with acute pulmonary embolism. *Reproduced with permission from Ref 16 (Yamasaki Y, et al. Dynamic Chest Radiography of Acute Pulmonary Thromboembolism. Radiology: Cardiothoracic Imaging 2022; 4(4):220086).

Supplementary file3 (MP4 23111 KB) Video 3. Dynamic perfusion images and pulmonary angiography of a 46-year-old man with chronic thromboembolic pulmonary hypertension before (a, b) and after (c, d) pulmonary endarterectomy. *Reproduced with permission from Ref 27 (Yamasaki Y, et al. A novel pulmonary circulation imaging using dynamic digital radiography for chronic thromboembolic pulmonary hypertension. Eur Heart J. 2020;41(26):2506).

Supplementary file4 (MP4 10812 KB) Video 4. Dynamic perfusion images of a 32-year-old man with acute pulmonary thromboembolism (a) and after six months of anticoagulation therapy (b). *Reproduced with permission from Ref 29 (Yamasaki Y, et al. Chronic thromboembolic pulmonary hypertension after acute pulmonary thromboembolism revealed by dynamic chest radiography. Eur Heart J Cardiovasc Imaging. 2022;23(6):e264-e265).

Supplementary file5 (MP4 19402 KB) Video 5. (a) Dynamic ventilation and (b) perfusion images of a 74-year-old man with severe left pulmonary artery stenosis caused by giant cell arteritis. *Reproduced with permission from Ref 31 (Yamasaki Y et al. Pulmonary ventilation-perfusion mismatch demonstrated by dynamic chest radiography in giant cell arteritis. Eur Heart J. 2021;42(2):208-209).

Supplementary file6 (MP4 6751 KB) Video 6. Movie of dynamic perfusion images (cross-correlation method) and invasive pulmonary angiography of a 52-year-old woman with a simple pulmonary arteriovenous malformation (one feeding artery and one drainage vein) before (a, b) and after (c, d) coil embolization of the feeding artery. *Reproduced with permission from Ref 7 (Yamasaki Y and Ishigami K. Dynamic chest Radiography of Pulmonary Arteriovenous Malformation. Radiology. 2021;300(2):285).
